# Three-dimensional microCT imaging of mouse heart development from early post-implantation to late fetal stages

**DOI:** 10.1007/s00335-022-09976-7

**Published:** 2023-01-03

**Authors:** Nanbing Li-Villarreal, Tara L. Rasmussen, Audrey E. Christiansen, Mary E. Dickinson, Chih-Wei Hsu

**Affiliations:** 1 Department of Integrative Physiology, Baylor College of Medicine, One Baylor Plaza, Houston, TX 77030 USA; 2 Cardiovascular Research Institute, Baylor College of Medicine, One Baylor Plaza, Houston, TX 77030 USA; 3 Department of Education, Innovation and Technology, Baylor College of Medicine, One Baylor Plaza, Houston, TX 77030 USA

## Abstract

Comprehensive detailed characterization of new mouse models can be challenging due to the individual focus involved in developing these models. Often models are engineered to test a specific hypothesis in a limited number of tissues, stages, and/or other contexts. Whether or not the model produces the desired phenotypes, phenotyping beyond the desired context can be extremely work intensive and these studies are often not undertaken. However, the general information resulting from broader phenotyping can be invaluable to the wider scientific community. The International Mouse Phenotyping Consortium (IMPC) and its subsidiaries, like the Knockout Mouse Project (KOMP), has made great strides in streamlining this process. In particular, the use of microCT has been an invaluable resource in examining internal organ systems throughout fetal/developmental stages. Here, we provide several novel vignettes demonstrating the utility of microCT in uncovering cardiac phenotypes both based on human disease correlations and those that are unpredicted.

## Introduction

The heart is the first organ to form during embryogenesis, and congenital heart defects are the most common birth defects, affecting about one percent of live human births. Additionally, ten percent or more of spontaneous gestational terminations are attributed to defects in cardiac development. Understanding the processes that go awry to cause these deaths is challenging to study directly in human patients. Animal models have provided critical insight into the regulatory networks that govern cellular processes such as specification, differentiation, migration, and proliferation that occur during cardiogenesis. In particular, mice have long been utilized to study human diseases including congenital heart disease.

The formation of the embryonic heart is remarkably similar between humans and mice. The earliest cardiac cells arise from the mesodermal cells that ingress through the primitive streak and migrate anteriorly to form the cardiac crescent during gastrulation stages. The cardiac crescent undergoes growth and morphological changes to form the linear heart tube, which subsequently loops and gives rise to the four-chambered heart. This process takes place in the span of 5 days in mice (embryonic day 7.5 (E7.5) to E12.5) and about 5 weeks in humans (week 3 to week 8). The embryonic heart is initially external and easily observed prior to and shortly after the mouse embryo turns around E9.0. By the time atrial and ventricular septation occurs (E11.5–E13.5), the embryonic heart is no longer easily observable making screening developmental defects difficult and requiring additional imaging methods for visualization and identification of potential heart defects.

High-resolution in situ imaging of embryonic heart development has been a challenge in the field for many years. Some imaging modalities, such as ultrasound or MRI, can be used to image the heart in intact embryos but provides lower resolution images (Petiet et al. 2008; Schneider et al. 2003; Smith 2001; Turnbull and Mori 2007; Zouagui et al. 2010). Other high-resolution imaging modalities such as high-resolution episcopic microscopy (HREM) (Mohun and Weninger 2011, 2012; Weninger et al. 2006) can provide detailed information about the development of the embryos at different stages. However, HREM is time consuming and is not wildly available. Iodine contrast microCT provides a platform for high -resolution imaging of both small and large sized samples, allowing detailed heart imaging in intact embryos at different stages in development without physically disrupting the embryos (Degenhardt et al. 2010; Metscher 2009a, b; Wong et al. 2012).

Many essential genes that regulate normal embryonic heart development have been well established in the past few decades. However, novel genes are still being identified either by correlation to human disease or by knockout studies in various model organisms. The International Mouse Phenotyping Consortium (IMPC) and its subsidiaries including the Knockout Mouse Project (KOMP/KOMP2) have made significant efforts to knockout as many single genes as possible in the mouse genome. To date (August 2022), this group has generated and characterized single knockout lines for 8267 genes. Of these lines, homozygotes for 784 die in utero (9.5%), including 705 lines that are homozygous lethal prior to E12.5 (mousephenotype.org). Therefore, 90% (705/784) of pre-natal lethal lines die by E12.5, which is the time point that the four-chambered heart should be formed and is required for proper oxygen and nutrient delivery to the embryo.

This effort by the IMPC and KOMP2 (NIH funded branch of the IMPC) has previously published that microCT is an exceptional platform for screening mutants in an unbiased way for internal morphological phenotypes (Dickinson et al. 2016; Hsu et al. 2019, 2016). This platform has been a rich resource for uncovering morphological cardiac defects in a high-throughput manner as part of a phenotyping pipeline to characterize each of the lethal knockout lines. We believe that it is also an extremely useful tool for examining individual mutant lines in a broad, unbiased manner. Here, we highlight several interesting cardiac phenotypes discovered in the Baylor College of Medicine (BCM) KOMP2 embryonic pipeline at early and late developmental stages. Some of these phenotypes would not have been discovered by observing gross morphology alone and due to pleiotropy, expressivity, and the prospect of section artifacts may not have been pinpointed at all by standard histological analysis.

## The progression of heart development during embryogenesis

**Fig. 1:**
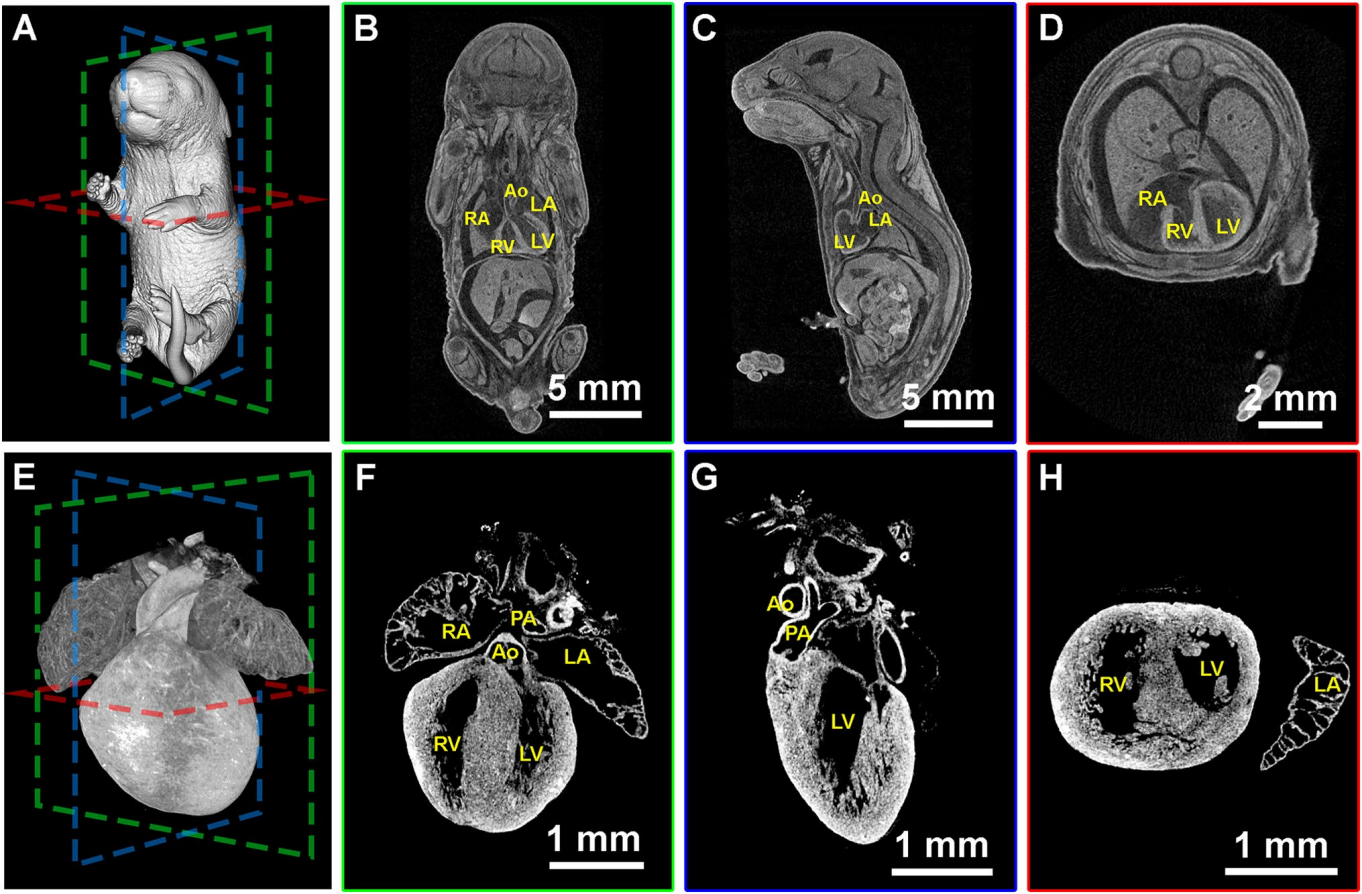
Iodine contrast microCT 3D imaging of mouse embryo (**A**–**D**) and heart (**E**–**H**) at E18.5. The 3D volumes of the E18.5 embryo and heart were rendered (**A** and **E**) and sectioned digitally at coronal (**B** and **F**), sagittal (**C** and **G**), and transverse (**D** and **H**) axis. *RA* right atrium. *LA* left atrium. *RV* right ventricle. *LV* left ventricle. *PA* pulmonary artery. *Ao* aorta

In late gestation stage embryos, visualization of the heart and other internal organs becomes a challenge as these organs are within the body cavity, skin has developed, and the embryo has lost translucency, making morphological examination without dissection impossible. However, by using iodine contrast microCT imaging, we and others are able to obtain 3D imaging data sets with resolution between 3 and 11 μm throughout gestational stages (Hsu et al. 2019, 2016; Metscher 2009a, b). An example of a wildtype (WT) E18.5 embryo imaged as part of the embryonic KOMP pipeline is shown in Fig. 1. After image reconstruction and post processing, we can visualize the 3D rendering of the whole embryo (Fig. 1A) or segmented regions, such as the heart (Fig. 1E). We can further digitally section through the entire embryo and the developing heart from coronal (Fig. 1B and and F), sagittal (Fig. 1C and G), and transverse planes (Fig. 1D and H). Being able to manipulate and rotate reconstructed embryos mitigates the need to precisely match mounting angles and sectioning depth necessary to collect matching sections of cardiac structures for immunostaining or histology. MicroCT also allows for image depth and resolution to obtain quality data from a range of sample sizes encompassing whole gastrulating embryos through whole neonatal pups, which is an improvement over other platforms such as HREM, OPT, and lightsheet.

**Fig. 2:**
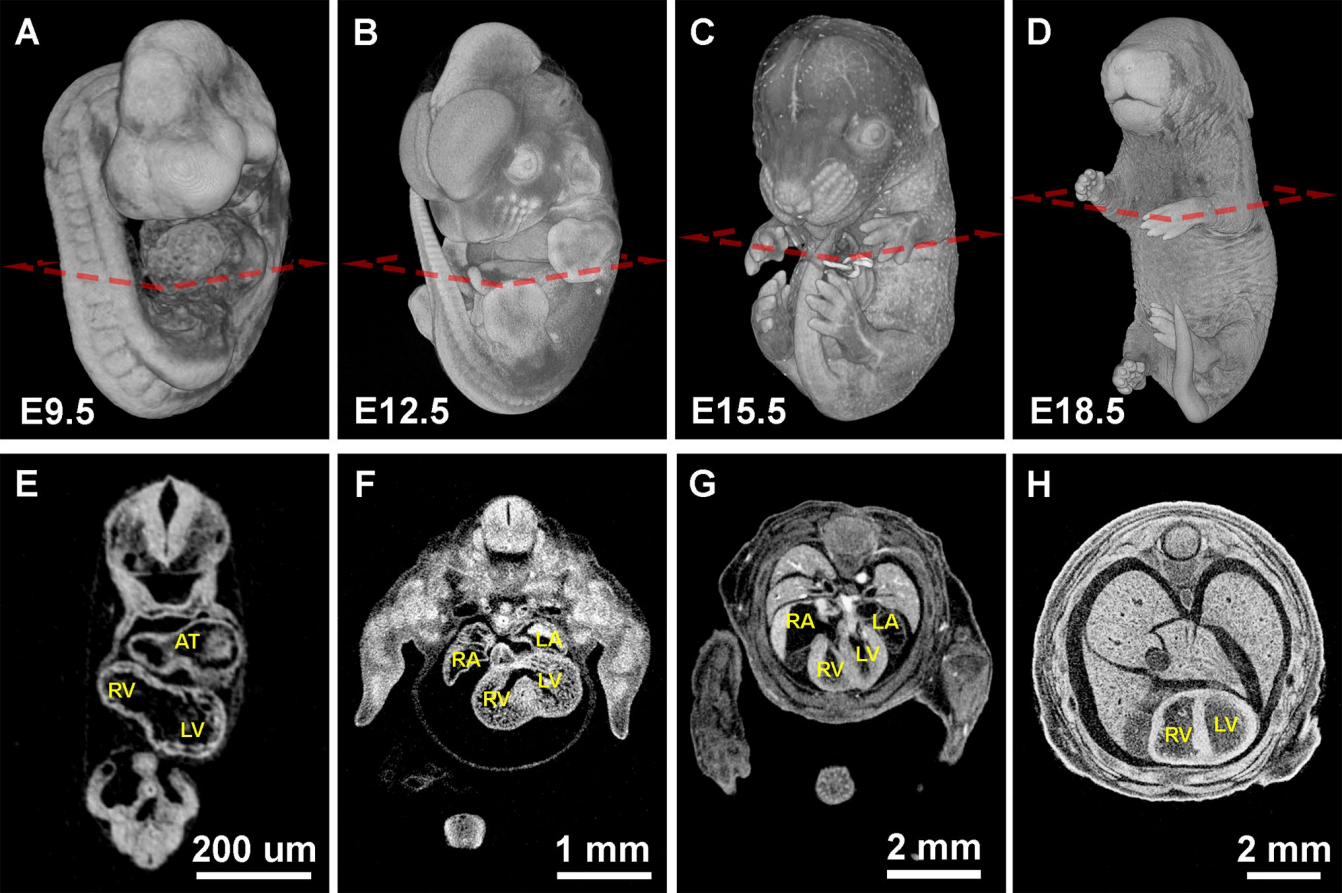
Mouse embryos from midgestation (E9.5) to late fetal (E18.5) stages imaged by iodine contrasted microCT. **A**–**D** Surface rendering of the mouse embryo at E9.5, E12.5, E15.5, and E18.5. **E**–**H** Digital transverse cross-sections show the progression of heart development. *AT* common atrium. *RA* right atrium. *LA* left atrium. *RV* right ventricle. *LV* left ventricle

MicroCT can also be utilized to visualize development of cardiac structures throughout embryogenesis. At E9.5, cardiac morphology is easily identifiable in whole mount renderings (Fig. 2A) and in digital sections (Fig. 2E). At this early stage of heart development, morphological defects such as hypertrophy, pericardial edema, and looping defects are also easy to see with light microscopy, while the embryo is relatively transparent, and the heart has not been internalized. However, internal defects at this stage, such as myocardial wall thickness and trabeculae formation, cannot be confidently identified without observing the internal structure of the developing heart by using histology or a 3D imaging technique, like microCT. Furthermore, by using microCT during later stages (Fig. 2B–D), additional internal structures such as valves, the septum, trabeculation, and vascular connections can be seen (Fig. 2F–H).

## Detection of cardiac defects at E9.5

**Fig. 3:**
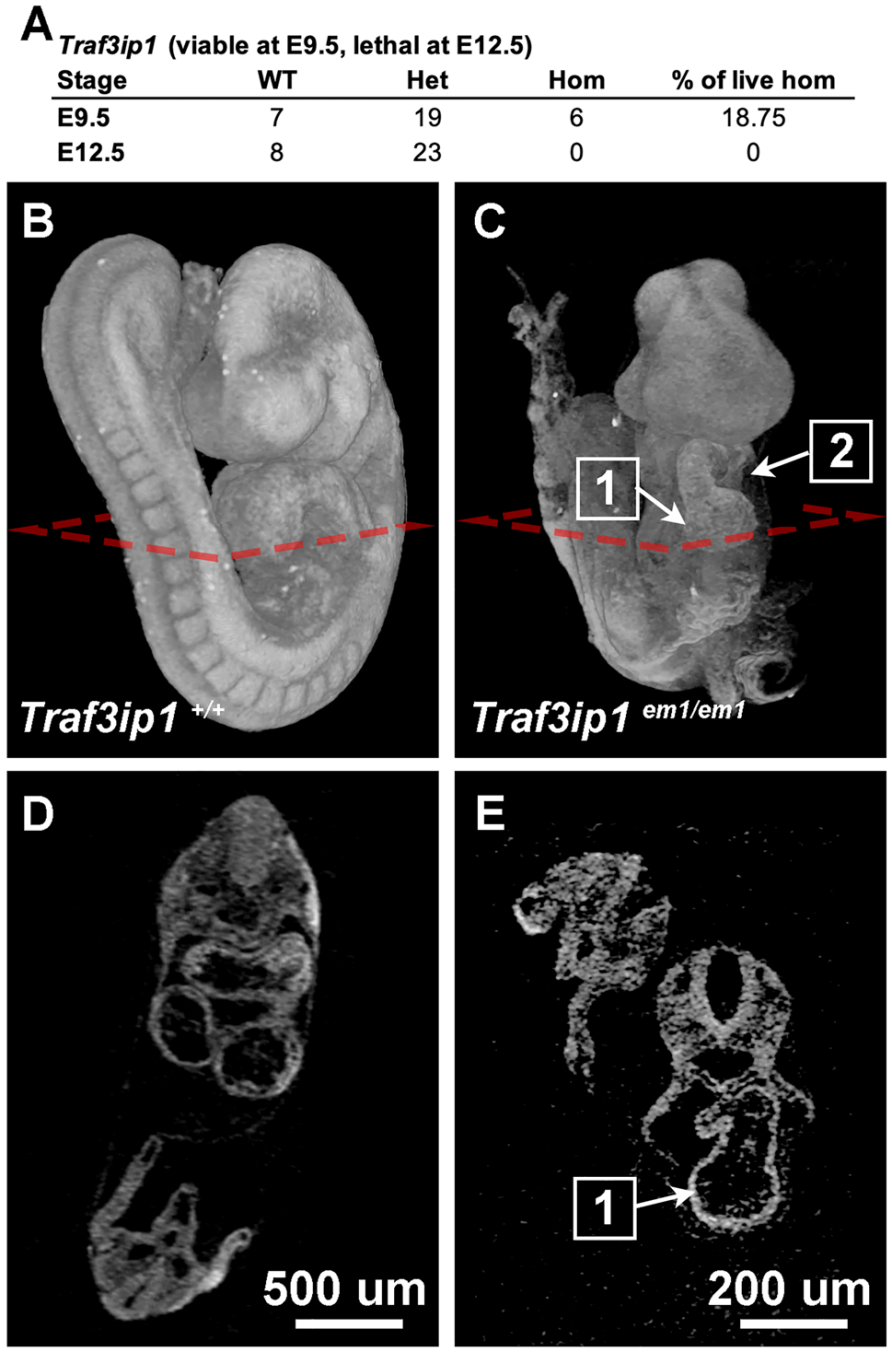
Cardiac defects identified in *Traf3ip1*^*em(IMPC)Bay*^. **A** Observed viability of *Traf3ip1*^*em(IMPC)Bay*^ at E9.5 and E12.5. MicroCT imaging of **B** and **D** WT and **C** and **E** *Traf3ip1* knockout embryos at E9.5. The *Traf3ip1* null embryo is unturned, with a (1) linear heart tube and (2) pericardium effusion

*Traf3ip1*^*em(IMPC)Bay*^ homozygous embryos show obvious defects in cardiac morphology at E9.5*.* Through the BCM KOMP2 embryonic pipeline, 6 live homozygous mutants were recovered out of 32 total embryos at E9.5, while zero out of 31 were recovered at E12.5 (Fig. 3A). By gross morphological analysis, littermate controls appear normal at E9.5 (Fig. 3B and D), while homozygous embryos for the *Traf3ip1* mutation are frequently delayed, unturned, and show abnormal somite morphology as well as abnormalities in heart tube looping and pericardial effusion, which can be seen in greater clarity by microCT (Fig. 3C and E). *Traf3ip1* encodes a subunit of the Intraflagellar Transport-B complex that is required for ciliogenesis. In humans, mutations in *TRAF3IP1* are associated with an autosomal recessive ciliopathy: Senior Loken Syndrome 9, which is ​​characterized by early-onset nephronophthisis and pigmentary retinopathy as well as liver defects, skeletal anomalies, and obesity (Bizet et al. 2015). In the 8 human patients described by Bizet, et al., one patient harbors a homozygous C-terminal frame shift mutation and the other patients harbor homozygous or heterozygous in-frame point mutations. Ciliopathies result from defects in formation and/or function of cilia, which are present in most cell types, can be motile or immotile/primary, and serve to generate directional flow for extracellular fluid or as molecular signaling hubs (Waters and Beales 2011). Cilia play a direct role in heart development as a hub for signaling pathways that are essential for regulating cardiovascular development including SHH, WNT, TGF-β, and BMP (Klena et al. 2017; Slough et al. 2008). In addition to its role in cilia formation, the protein encoded by *Traf3ip1* functions in regulating cytosolic microtubule stability via a conserved tubulin binding domain (Bizet et al. 2015). The previously described *Traf3ip1 mutant* mouse has neural tube defects, polydactyly and does not develop past E13.5 (Berbari et al. 2011). This mutant has a gene trap insertion in intron 11, which may allow for a stable hypomorphic protein. The *Traf3ip1*^*em(IMPC)Bay*^ mouse described here has a CRISPR mediated 2.3 kb deletion that includes exons 6 and 7 and is predicted to result in a frameshift mutation and nonsense mediated decay. The mutations in the other mouse model and those found in human patients are likely hypomorphic, allowing for partial gene functions and resulting in milder phenotypes. Although cilia are important in proper heart development and function, the role of Traf3ip1 in human heart disease has yet to be identified. These data suggest that the role of Traf3ip1 in murine and human cardiac development deserves further exploration.

**Fig. 4:**
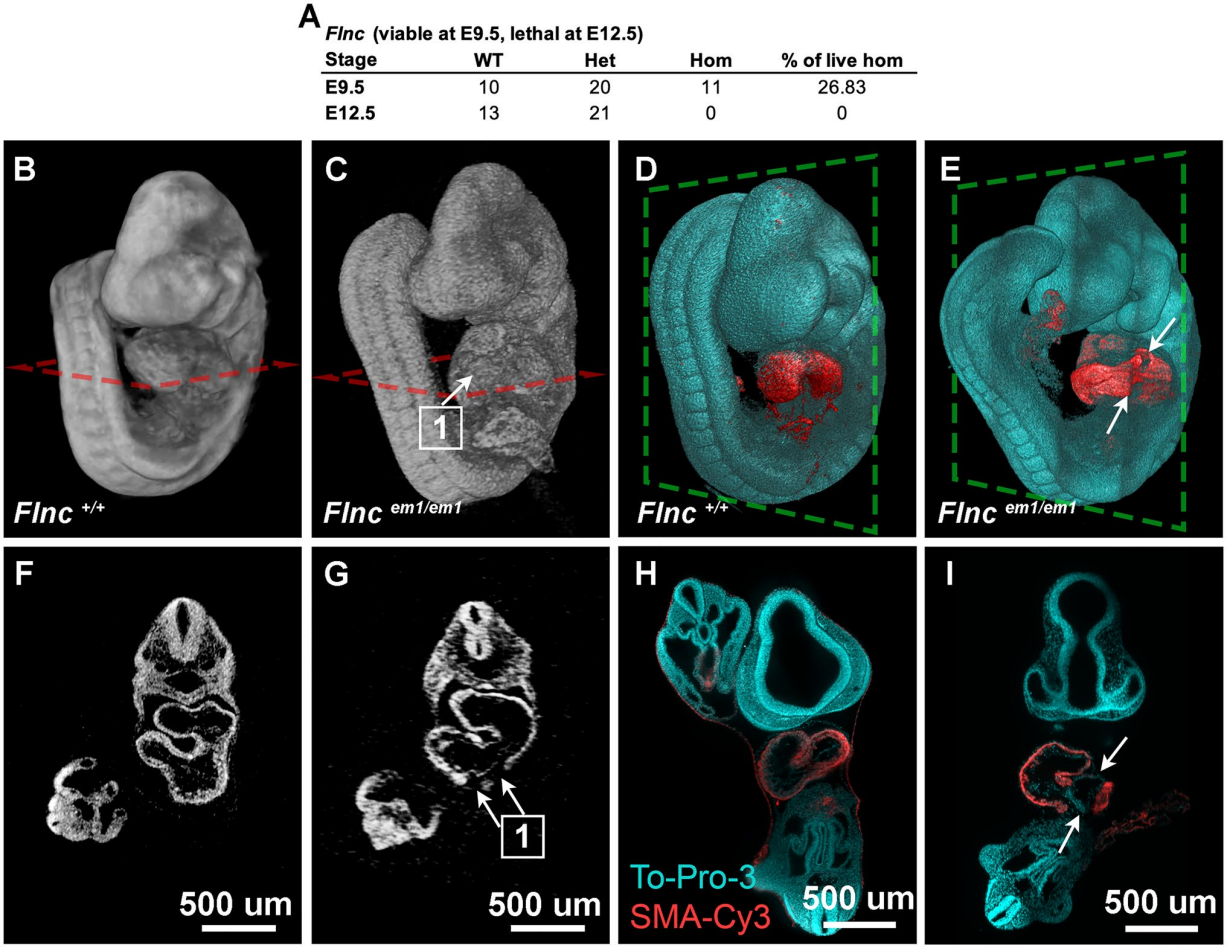
Cardiac defect in *Flnc*^*em(IMPC)Bay*^. **A** Observed viability of *Flnc*^*em(IMPC)Bay*^ at E9.5 and E12.5. MicroCT imaging of **B** and **F** WT and **C** and **G** *Flnc* knockout embryos at E9.5. The *Flnc* null embryo shows (1) breaks in the pericardium wall from iodine contrasted microCT (**C** and **G**). Lightsheet imaging of the cleared (**D** and **H**) WT and (**E** and **I**) *Flnc* knockout embryos stained with To-Pro-3 (cyan) and smooth muscle alpha actin conjugated with Cy3 (red) confirmed the break (white arrows) in the pericardium wall of (**E** and **I**) *Flnc* null embryo

The *Flnc*^*em(IMPC)Bay*^ line is another good example of a line with homozygous embryos that have early cardiac phenotypes that can be resolved by microCT. At E9.5, 11 live homozygous *Flnc* mutants were recovered from 41 total embryos. At E12.5, zero out of 34 live homozygous mutants were recovered from the KOMP screen (Fig. 4A). *Flnc* mutant embryos at E9.5 were found to have abnormal heart morphology with irregular heart looping, poor blood flow, pericardial edema, and unusual opaque spots in the endocardium by gross morphology under a stereo microscope. The protein encoded by *Flnc* is an actin binding structural protein whose variants are associated with cardiac and muscular phenotypes and is a prevalent contributor to genetic dilated cardiomyopathies (DCM) in human patients (Verdonschot et al. 2020). DCM is one of the leading causes of heart failure and is characterized by the thinning and stretching of the left ventricle resulting in ventricular dilation and reduced contractile function (Reichart et al. 2019). Close examination of reconstructed microCT images shows a break in the myocardial wall of *Flnc* mutants (Fig. 4G) not seen in WT embryos (Fig. 4F). Although the heart and its development are clearly abnormal compared to litter mate controls seen by 3D rendering of the microCT data (Fig. 4B–C), the opaque spots are an unusual phenotype that could not be explained without further investigation. microCT sections show what appeared to be breaks in the myocardium of the mutant (Fig. 4G) that are not observed in controls (Fig. 4F). This phenotype was confirmed by whole mount smooth muscle actin (SMA) immunostaining at E9.5 followed by lightsheet microscopy. Although SMA is broadly thought of as a smooth muscle marker, very little smooth muscle has formed by E9.5 and it is expressed in cardiac muscle at this stage (Clement et al. 2007). While SMA expression is detected in a continuous loop around the wildtype embryonic heart (Fig. 4D and H), SMA expression has multiple breaks in the *F*lnc mutant hearts (Fig. 4E and I). This confirms that the lack of contrast observed in the microCT is in fact due to a myocardial wall defect. Therefore, even at early stages, microCT is a useful imaging platform for screening novel lines. The phenotypes discovered can also be further verified and enhanced using traditional methods.

## MicroCT detects cardiac phenotypes in late stage mouse embryos

**Fig. 5:**
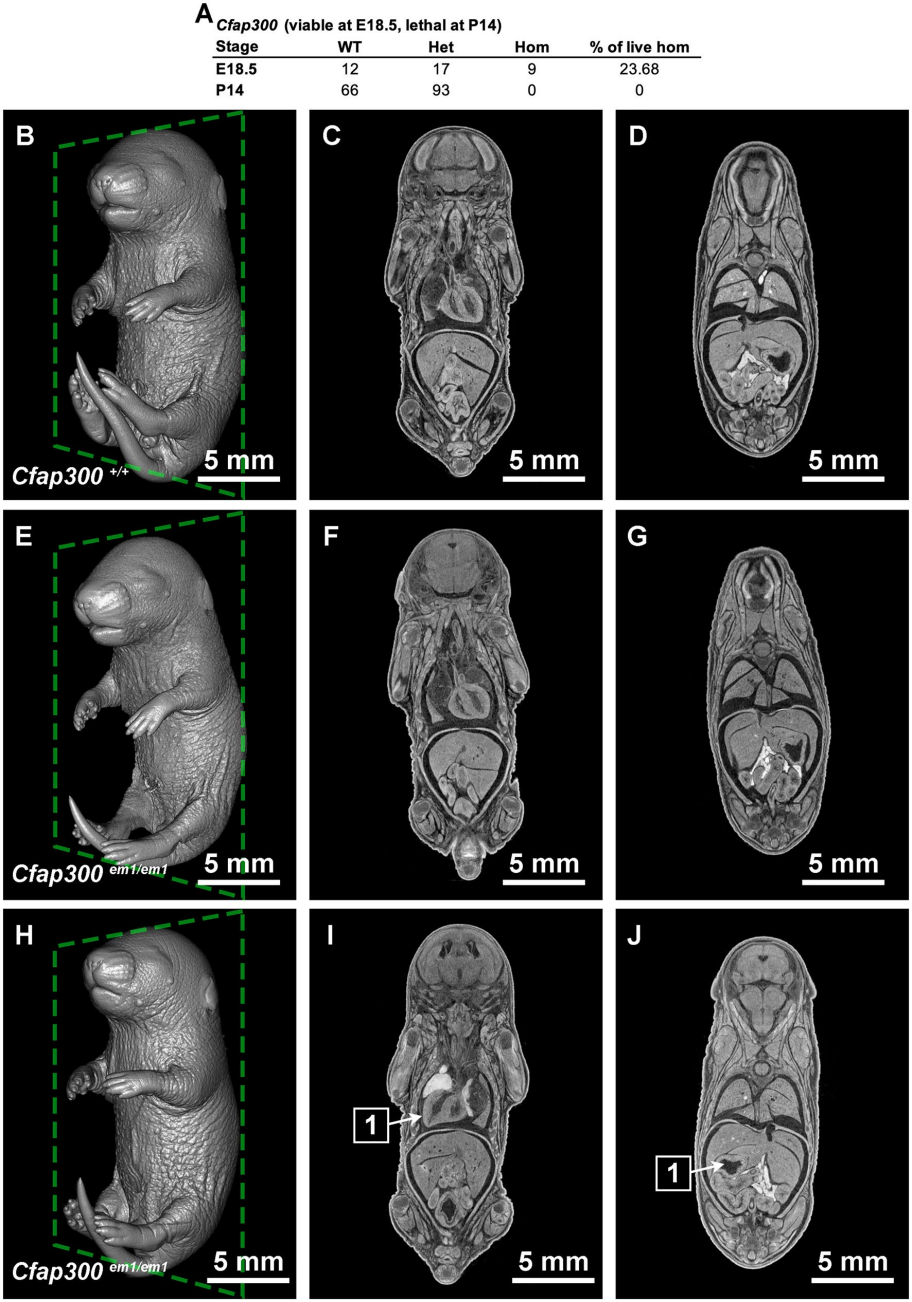
Cardiac defect in *Cfap300*^*em(IMPC)Bay*^. **A** Observed viability of *Cfap300*^*em(IMPC)Bay*^ at E18.5 and P14. MicroCT imaging of (**B**–**D**) WT and *Cfap300* knockout embryos that is **E**–**G** situs solitus or **H**–**J** situs inversus at E18.5. 1 arrows point to inverted **I** heart and **J** stomach

Detection of novel cardiac phenotypes by microCT is even more powerful in late stage embryos because the heart is internalized. For example, mutations in *CFAP300* have been associated with Primary Ciliary Dyskinesia (PCD) (Fassad et al. 2018; Zietkiewicz et al. 2019). Patients with PCD have chronic respiratory symptoms and distress as well as possible deafness and situs inversus. Interestingly, mice generated by the BCM KOMP2 pipeline harboring the homozygous *Cfap300*^*em(IMPC)Bay*^ allele were found to be viable at E18.5, but lethal at postnatal day 14 (P14) (Fig. 5A). Upon examination, 11 homozygous fetuses were identified at E18.5. Two of these were dead and showed signs of widespread edema and hemorrhaging. The remaining 9 were unremarkable for gross morphology compared to WT. Seven of the live homozygotes were imaged by microCT. Of these, 3 appeared normal (situs solitus) and 4 had situs inversus, 2 of which were accompanied by hydrocephaly. The 3D rendering is shown for a WT littermate (Fig. 5B–D), a situs solitus homozygous fetus (Fig. 5E–G) and a situs inversus homozygous fetus (Fig. 5H–J).

Situs inversus is a reversal of the typical left right asymmetry (situs solitus), where the heart loops to the right, the stomach and spleen are found on the left side, and the liver and gall bladder are found on the right. Left right asymmetry has been shown to be regulated by the directionality of currents created by moving cilia (Pennekamp et al. 2015). Ciliopathy often results in a lack of cilia motion and therefore no current. In these conditions, it has been shown that the directionality is stochastic, meaning that the asymmetrical organ morphogenesis still happens, but that the directionality is no longer regulated, so 50% of embryos develop with situs solitus and 50% develop with situs inversus. This ratio fits with the observation that 3/7 fetuses had situs solitus (Fig. 5F–G) and 4/7 microCT imaged E18.5 fetuses had situs inversus (Fig. 5I–J). While we cannot assess whether the animals have hearing loss or respiratory symptoms by imaging of fixed samples, the morphological changes observed suggest that the *Cfap300* mutant mice are mimicking human PCD. This partially penetrant phenotype can be difficult to observe using standard histology because it is work intensive to embed and cut large numbers of tissues from controls and mutants to analyze and assess the phenotype. Samples could be imaged using lightsheet or OPT; however, these technologies have a large trade off in getting the samples optically transparent in order to gain the working depth of a large tissue like an E18.5 whole mouse embryo. Using microCT, we can image late stage embryos in entirety in 2 h per embryo and identify these partially penetrant phenotypes.

**Fig. 6:**
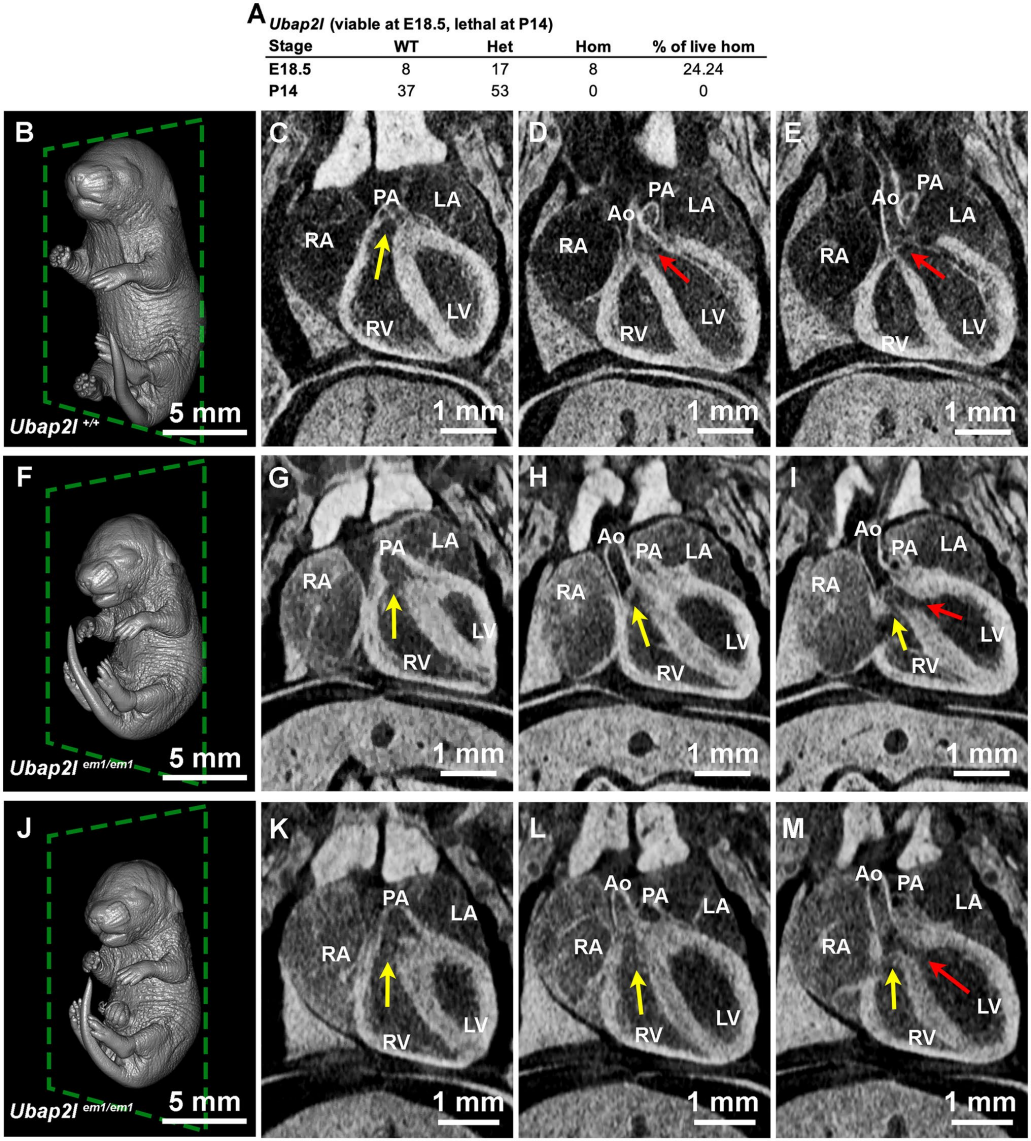
Cardiac defect in *Ubap2l*^*em(IMPC)Bay*^. **A** Observed viability of *Ubap2l*^*em(IMPC)Ba*^ at E18.5 and P14. MicroCT imaging of **B**–**E** WT and **F**–**M** *Ubap2l* knockout embryos with double outlet right ventricles (DORV). Yellow arrow shows the blood flow from the right ventricles, and red arrow shows the blood flow direction from the left ventricle. *RA* right atrium. *LA* left atrium. *RV* right ventricle. *LV* left ventricle. *PA* pulmonary artery. *Ao* aorta

Some single gene mouse knockouts have phenotypes that would not be predicted based on human disease. For example, *Ubap2l*^*em(IMPC)Bay*^ is another line generated by the BCM KOMP2 pipeline. UBAP2L is a BMI1-interacting protein essential for hematopoietic stem cell activity (Bordeleau et al. 2014). *Ubap2l* is amplified in a large subset of human lung adenocarcinoma and is critical for epithelial lung cell identity and tumor metastasis (Aucagne et al. 2017). Interestingly, this single gene mouse knockout is lethal by P14 (0/90) (Fig. 6A). Eight E18.5 homozygous fetuses were obtained. Of these, 6 were screened by microCT. While the littermate controls appeared normal (Fig. 6B–E), all six homozygous embryos were smaller (Fig. 6F and J), had hydrocephaly (data not shown), and were found with double outlet right ventricles (DORV) (Fig. 6G-I, K–M). In both examples, the right ventricle (RV) has an outlet to the pulmonary artery (PA) (Fig. 6 G and K) similar to the wildtype control (Fig. 6C). However, unlike the WT control (Fig. 6D and E), the RV of the mutant fetuses also empties into the aorta (Ao) (Fig. 6 H and L) and/or mixes with the blood in the left ventricle (LV) (Fig. 6I and M). DORV is a congenital heart disease where the RV leads to both the aorta and the pulmonary artery instead of the RV leading to the pulmonary artery and the LV leading to the aorta. This phenotype is difficult to detect without imaging the heart within the thoracic chamber because once vessels are detached, it is challenging to distinguish them from one another. Furthermore, obtaining histological sections in which the lumen of the pulmonary artery and dorsal aorta are clearly attaching to and emptying into a chamber is a demanding task dependent on sectioning blindly through the required angle. 3D volumes of the entire embryo obtained from microCT allow for vessels to be traced back to their origins, and angles of slices can be adjusted to visualize these morphological anomalies. Here, we have detected an unexpected cardiac phenotype in a novel single gene knockout model which deserves further exploration.

## Conclusions

We have highlighted four vignettes in which the BCM KOMP2 pipeline has further defined embryonic cardiac phenotypes using microCT as a tool. MicroCT has been extremely useful for this high-throughput “pipeline” type study. Many interesting and relevant phenotypes could be overlooked using work-intensive or biased approaches, such as performing histology and imaging only at the organs of interest.

We have highlighted examples for early post-implantation (E9.5) and late stage (E18.5) embryos to emphasize that the microCT platform delivers more than sufficient imaging capacity and resolution for this range of tissue sizes. Also, it is in the nature of our pipeline to image at the last viable stage. Interestingly alleles that have cardiac defects frequently die by E12.5. If they survive this midgestation stage, they can typically survive until birth. The models described at E18.5 could easily be imaged at earlier stages, but this time-course analysis is not in the scope of the BCM KOMP2 pipeline. We advocate for broader use of this unbiased imaging tool when groups are characterizing new mouse models and alleles.

## Data Availability

The data analyzed during the current study are available from the corresponding author on reasonable request.
